# Overexpression of the Golden SNP-Carrying *Orange* Gene Enhances Carotenoid Accumulation and Heat Stress Tolerance in Sweetpotato Plants

**DOI:** 10.3390/antiox10010051

**Published:** 2021-01-04

**Authors:** So-Eun Kim, Chan-Ju Lee, Sul-U Park, Ye-Hoon Lim, Woo Sung Park, Hye-Jin Kim, Mi-Jeong Ahn, Sang-Soo Kwak, Ho Soo Kim

**Affiliations:** 1 Plant Systems Engineering Research Center, Korea Research Institute of Bioscience and Biotechnology (KRIBB), 125 Gwahak-ro, Daejeon 34141, Korea; sonic0312@kribb.re.kr (S.-E.K.); moda22@kribb.re.kr (C.-J.L.); sulu0849@kribb.re.kr (S.-U.P.); lim0916@kribb.re.kr (Y.-H.L.); 2 Department of Environmental Biotechnology, KRIBB School of Biotechnology, University of Science and Technology (UST), 217 Gajeong-ro, Daejeon 34113, Korea; 3 College of Pharmacy and Research Institute of Life Sciences, Gyeongsang National University, 501 Jinjudae-ro, Jinju 52828, Korea; pws8822@gmail.com (W.S.P.); black200203@gmail.com (H.-J.K.); amj5812@gnu.ac.kr (M.-J.A.)

**Keywords:** sweetpotato, carotenoid, orange, IbOr, IbOr-R96H

## Abstract

Carotenoids function as photosynthetic accessory pigments, antioxidants, and vitamin A precursors. We recently showed that transgenic sweetpotato calli overexpressing the mutant sweetpotato (*Ipomoea batatas* [L.] Lam) *Orange* gene (*IbOr*-*R96H*), which carries a single nucleotide polymorphism responsible for Arg to His substitution at amino acid position 96, exhibited dramatically higher carotenoid content and abiotic stress tolerance than calli overexpressing the wild-type *IbOr* gene (*IbOr*-*WT*). In this study, we generated transgenic sweetpotato plants overexpressing *IbOr*-*R96H* under the control of the cauliflower mosaic virus (CaMV) *35S* promoter via *Agrobacterium*-mediated transformation. The total carotenoid contents of *IbOr*-*R96H* storage roots (light-orange flesh) and *IbOr*-*WT* storage roots (light-yellow flesh) were 5.4–19.6 and 3.2-fold higher, respectively, than those of non-transgenic (NT) storage roots (white flesh). The β-carotene content of *IbOr*-*R96H* storage roots was up to 186.2-fold higher than that of NT storage roots. In addition, *IbOr*-*R96H* plants showed greater tolerance to heat stress (47 °C) than NT and *IbOr*-*WT* plants, possibly because of higher DPPH radical scavenging activity and ABA contents. These results indicate that *IbOr*-*R96H* is a promising strategy for developing new sweetpotato cultivars with improved carotenoid contents and heat stress tolerance.

## 1. Introduction

Given their functional importance, carotenoids have been investigated since the beginning of the 19th century, and over 1100 carotenoid compounds have been identified to date [1]. Tetraterpenoids are lipid-soluble carotenoid pigments produced by all photosynthetic organisms, including plants, algae, bacteria, and fungi. In plants, carotenoids are generally categorized into two classes, xanthophylls, and carotenes. These carotenoids are red, orange, and yellow-colored pigments, which are responsible for the coloration of fruits and flowers. These carotenoids also play a vital role in protecting the photosynthetic apparatus from photo-oxidation, which can trigger the formation of reactive oxygen species (ROS) [2,3]. In addition to their functions in photosynthetic organisms, carotenoids also serve as precursors of pro-vitamin A, and exhibit antioxidant properties and the ability to relieve chronic diseases in humans and animals [4,5]. In developing countries, vitamin A deficiency affects approximately 130 million preschool-aged children and 7 million pregnant women and is responsible for the death of 0.6 million children aged under 5 years [6]. To alleviate global health issues caused by the lack of micronutrients, several studies have been carried out to investigate the biofortification of plant-derived foods with carotenoid precursors of pro-vitamin A (mainly β-carotene); for example, Golden Rice, which contains a high level of carotenoids [7,8]. These studies have led to the development of a number of biofortified crops using transgenic approaches by exploiting genes involved in the carotenogenesis pathway [9,10].

Sweetpotato (*Ipomoea batatas* [L.] Lam) is the seventh most important food crop in the world and a rich source of diverse nutrients [11]. It is believed that sweetpotato, especially orange-fleshed cultivars, could combat vitamin A deficiency because of the high level of β-carotene (approximately 90% of all carotenoids) [12]. Sweetpotato has a high nutritional value and is rich in low molecular weight antioxidants (LMWAs) such as carotenoids, ascorbate, tocopherols, and polyphenols [13,14]. Sweetpotato is also considered a valuable source of unique natural products, including some that can be used for the development of medicines effective against various diseases and for the production of industrial products [15,16]. To develop new sweetpotato cultivars with high LMWA content, genetic engineering of the biosynthetic and metabolic genes of LMWAs is currently in progress via molecular breeding. Metabolic engineering of carotenoid biosynthesis genes has been investigated to increase the accumulation of different types of carotenoids. Transgenic sweetpotato plants with enhanced carotenoid content also exhibit environmental stress tolerance [13,14].

The *Orange* (*Or*) gene, which encodes the plastid-localized cysteine-rich DnaJ protein, regulates carotenoid accumulation and abiotic stress resistance in various plant species. Ectopic expression of *Or* promotes carotenoid accumulation in various crop species [17,18,19,20,21,22,23,24,25]. In sweetpotato, the IbOr protein functions as a holdase chaperone, a post-transcriptional regulator of phytoene synthase (PSY), a rate-limiting enzyme in the carotenoid biosynthetic pathway [21,26]. Furthermore, IbOr interacts with oxygen-evolving enhancer protein 2-1 (PsbP) in the chloroplast, a member of the protein complex in photosystem II (PSII), and protects it from heat-induced denaturation [26,27]. IbOr also interacts with carotenoid cleavage dioxygenase 4 (IbCCD4) in the chloroplast, which is consistent with the location of the interaction of IbOr with IbPSY and IbPsbP. The interaction of IbOr and IbCCD4 does not affect the cleavage of β-carotene [28]. Thus, IbOr is a multifunctional protein that exhibits strong holdase chaperone activity and contributes to carotenoid accumulation and abiotic stress tolerance in plants.

Recently, a dominant or allele carrying a single nucleotide polymorphism (SNP; referred to as the golden SNP) was identified in melon (*Cucumis melo*); this SNP causes arginine (Arg) to histidine (His) substitution in the CmOr protein and is associated with high β-carotene accumulation in melon [18]. Overexpression of AtOr-His, a variant of the *Arabidopsis thaliana* or gene carrying the golden SNP, in *Arabidopsis* calli and tomato (*Solanum lycopersicum*), leads to high-level accumulation of carotenoids [22,29]. In addition, the other SNP was identified between low- and high-carotenoid genotypes in carrot (*Daucus carota*); this transition of T to C results in a change of the codon TTG to TCG, causing SNP of leucine (Leu) to serine (Ser) and is related to high β-carotene accumulation in carrot [30]. Recently, we performed site-directed mutagenesis of the *IbOr* gene, leading to Arg to His substitution at position 96 (based on the CmOr protein) in the IbOr protein (IbOr-R96H), and generated *IbOr*-*R96H* transgenic sweetpotato calli. Transgenic calli overexpressing *IbOr*-*R96H* showed significantly higher carotenoid contents and enhanced salt and heat stress tolerance than those overexpressing the wild-type *IbOr* (*IbOr*-*WT*) gene [31]. In this study, we generated transgenic sweetpotato plants overexpressing *IbOr*-*R96H* to study its effect at the whole-plant level. Our results showed that overexpression of *IbOr*-*R96H* increased the content of total carotenoids, especially β-carotene, in storage roots of transgenic sweetpotato plants. In addition, we investigated abiotic stress tolerance assay using leaf discs and monitored transcript levels of carotenoid biosynthesis and degradation genes. Interestingly, *IbOr*-*R96H* transgenic plants exhibited high DPPH radical scavenging activity in both fresh storage roots and leaves compared with non-transgenic (NT) and *IbOr*-*WT* plants.

## 2. Materials and Methods

### 2.1. Plant Materials and Growth Conditions

A white-fleshed sweetpotato cultivar, Xushu 29, was kindly provided by the Sweetpotato Research Institute, Chinese Academy of Agricultural Science, Xuzhou, China. Plants were grown in a growth chamber under controlled conditions (25 ± 3 °C temperature, under a light intensity of 150 μmol m^−2^ s^−1^ and 16 h light/8 h dark photoperiod). To analyze carotenoid content, the storage roots of sweetpotato plants were harvested 4 months after planting.

### 2.2. Plasmid Construction and Agrobacterium-Mediated Transformation

Site-directed mutagenesis of *IbOr* was performed as described previously [31]. *IbOr*-*WT* (accession no. HQ828087) and *IbOr*-*R96H* were cloned into the pGWB5 binary Gateway vector under the control of the cauliflower mosaic virus (CaMV) *35S* promoter to generate fusions with the *GFP* gene. The constructs were transformed into sweetpotato embryogenic calli using *Agrobacterium tumefaciens* strain GV3101 [23].

### 2.3. Generation of Transgenic Sweetpotato Plants

Transgenic plants were first screened on hygromycin-containing medium and then confirmed by genotyping and quantitative real-time PCR (qRT-PCR), which were performed using genomic DNA and RNA extracted from fresh leaf samples (0.1 g) of hygromycin-resistant sweetpotato plants. Genomic DNA was amplified using the following primers: 5′-CGCACAATCCCACTATCCTT-3′ and 5′-TTCCCAAGCTCAGCATTCTT-3′. To analyze the expression of *IbOr*-*R96H*, qRT-PCR was performed using the following primers: 5′-CTTCATCCGTCGACACCGAA-3′ and 5′-GCTGTATTCTCAGCCTACGA-3′.

### 2.4. RNA Extraction and qRT-PCR Analysis of Carotenogenesis-Related Genes

Total RNA was extracted from the leaves and storage roots of 3-week-old and 4-month-old transgenic sweetpotato plants, respectively, using the method reported previously [31]. To analyze the expression of sweetpotato genes involved in carotenoid biosynthesis and degradation pathways, qRT-PCR was performed on a CFX96 Touch™ Real-Time PCR Detection System (Bio-Rad, Hercules, CA, USA), according to the manufacturer’s instructions, using EvaGreen fluorescent dye and gene-specific primers (Tables S1 and S2).

### 2.5. Analysis of Carotenoid Contents

Carotenoids were extracted from storage roots of 4-month-old transgenic sweetpotato plants using acetone (0.01% BHT). The extracted carotenoids were analyzed using the Agilent 1260 high-performance liquid chromatography (HPLC) system (Hewlett-Packard, Waldbronn, Germany), as described previously [32]. All extraction procedures were conducted under dim light to avoid pigment degradation and loss. Carotenoid contents were expressed as μg g^−1^ dry weight (DW) of storage roots.

### 2.6. Analysis of Heat Tolerance

To induce heat stress, leaf discs (12 mm in diameter) were excised from the 3rd, 4th, and 5th leaves of 1-month-old transgenic and NT sweetpotato plants, and exposed to high temperature (47 °C) in the dark for 12 h. To visualize the degree of damage caused by heat stress, heat-treated leaf discs were floated on 3,3′-diaminobenzidine (DAB) solution (1 mg mL^−1^ in water; pH 3.8), as described previously [27]. To measure the extent of cellular damage or membrane disruption, ion leakage was quantified using an ion conductivity meter (MTD, Schwerzenbach, Switzerland).

### 2.7. Determination of ABA Content

ABA was extracted from the leaf discs of individual plants and its amount was determined with a plant hormone abscisic acid (ABA) ELISA Kit (MyBioSource Inc., San Diego, CA, USA) following manufacturer instruction. Absorbance at 450 nm was detected using Bio-Rad i-Mark Microplate Reader (Bio-Rad). Three independent experiments were performed.

### 2.8. Antioxidant Activity Analysis

The analysis of 2,2-diphenyl-1-picrylhydrazyl (DPPH) radical scavenging activity is a widely used spectrophotometric methods for the assessment of the antioxidant capacities inhibiting lipid oxidation in complex biological mixtures such as plant or food extracts. The assay was analyzed as described previously [33]. Leaf and storage root extracts of transgenic and NT sweetpotato plants were prepared in methanol, and the absorbance of the extracts was determined at 517 nm using a spectrophotometer. A calibration curve was constructed using L-Ascorbic acid (AsA) as a standard, and the DPPH radical scavenging activity of the extracts was calculated as AsA equivalents per gram of tested samples.

### 2.9. Statistical Analysis

Data were subjected to one-way analysis of variance (ANOVA). Significant differences were identified by Tukey’s honestly significant difference (HSD) post hoc test at *p* < 0.05.

## 3. Results

### 3.1. IbOr-R96H Transgenic Sweetpotato Plants Exhibited a Light-Orange Color

**Figure 1:**
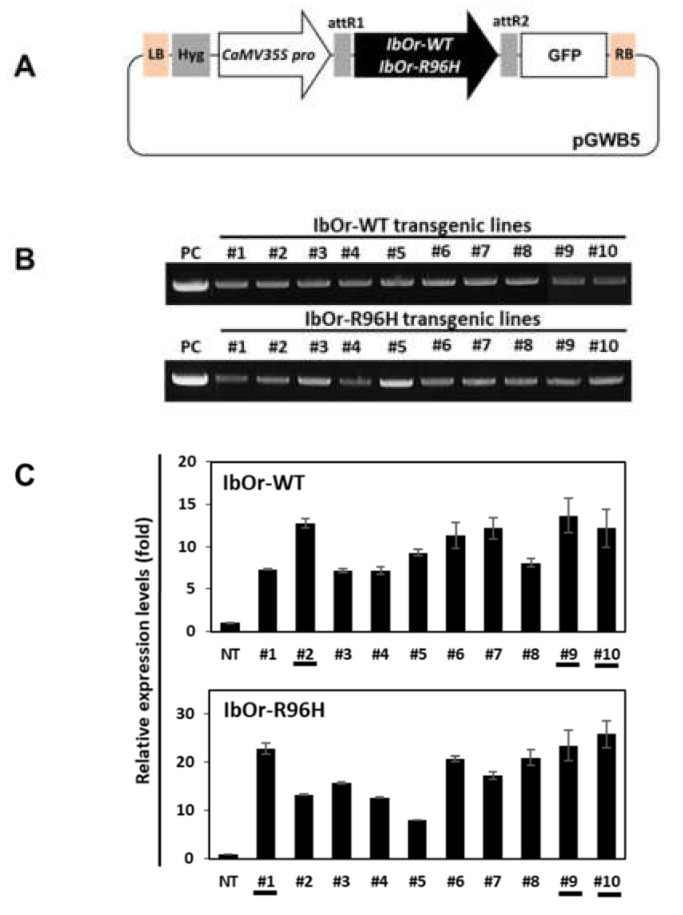
Generation of transgenic sweetpotato plants overexpressing *IbOr*-*WT* and *IbOr*-*R96H*. (**A**) Schematic representation of the constructs used for the production of transgenic sweetpotato plants overexpressing *IbOr*-*WT* and *IbOr*-*R96H*. (**B**) Genotyping transgenic plants using *IbOr*-specific primers. PC, positive control. (**C**) Transcript levels of *IbOr* in transgenic sweetpotato plants. Data represent the mean ± standard deviation (SD) of three technical replicates.

Previously, we reported that overexpression of *IbOr*-*R96H* dramatically increased the carotenoid content of transgenic sweetpotato calli compared with WT sweetpotato calli. The total carotenoid and β-carotene contents of *IbOr*-*R96H* transgenic sweetpotato calli with dark-orange flesh were 13.3- and 39.3-fold higher, respectively, than those of *IbOr*-*WT* calli. Moreover, transgenic calli also showed increased tolerance to salt stress (150 mM NaCl) and heat stress (47 °C) [31]. To examine whether IbOr-R96H is functionally conserved in sweetpotato calli and plants, *IbOr*-*WT* and *IbOr*-*R96H* were overexpressed under the control of the constitutive CaMV *35S* promoter in the embryogenic callus of sweetpotato (cv. Xushu 29) using *Agrobacterium*-mediated transformation (Figure 1A). Ten independent transgenic lines were generated for each construct and confirmed by genomic DNA-based PCR using construct-specific primers (Figure 1B). Expression levels of *IbOr*-*WT* and *IbOr*-*R96H* were evaluated in each transgenic line using qRT-PCR, and three lines per construct (*IbOr*-*WT* [#2, #9, #10] and *IbOr*-*R96H* [#1, #9, #10]), showing high *IbOr* transcript levels, were selected for further characterization (Figure 1C).

**Figure 2:**
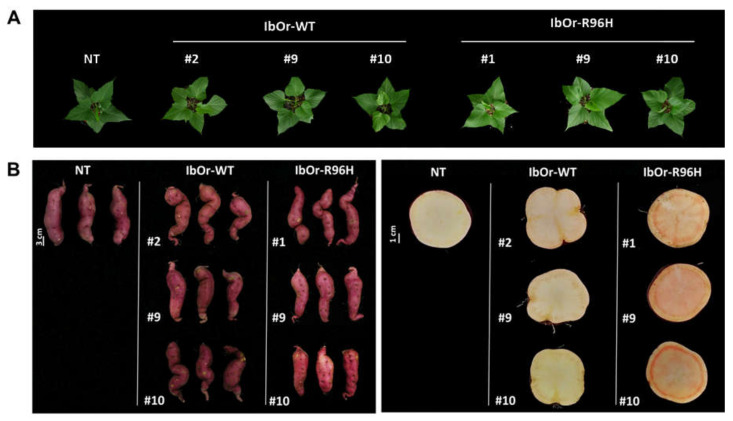
Phenotypic analysis of transgenic sweetpotato plants overexpressing *IbOr*-*WT* and *IbOr*-*R96H*. (**A**) Photographs of aerial plant parts of 1-month-old NT, *IbOr*-*WT*, and *IbOr*-*R96H* plant lines. (**B**) Comparison of storage roots of 4-month-old pot-grown NT, *IbOr*-*WT*, and *IbOr*-*R96H* plants. Photographs show whole storage roots (left) and cross-sections of storage roots (right).

To determine whether the overexpression of *IbOr*-*R96H* increased carotenoid synthesis in transgenic sweetpotato plants, we first evaluated the morphological phenotypes of NT, *IbOr*-*WT*, and *IbOr*-*R96H* plants. Stem cuttings of NT, *IbOr*-*WT* (#2, #9, #10), and *IbOr*-*R96H* (#1, #9, #10) plants were grown in plastic pots for 4 months. One-month-old plants of transgenic *IbOr*-*WT* and *IbOr*-*R96H* lines exhibited no significant phenotypic alterations in aerial plant parts compared with NT plants (Figure 2A). However, the skin of *IbOr*-*R96H* transgenic storage roots exhibited a light-red color compared with that of NT and *IbOr*-*WT* storage roots (Figure 2B, left). Interestingly, storage roots of *IbOr*-*R96H* plants exhibited a light-orange color overall and a dark-orange color around the cambium rings, whereas storage roots of *IbOr*-*WT* plants showed a deeper yellow color around the cambium layers than those of NT plants (Figure 2B, right).

### 3.2. IbOr-R96H Overexpression Induces Overaccumulation of Carotenoids in Storage Roots

**Figure 3:**
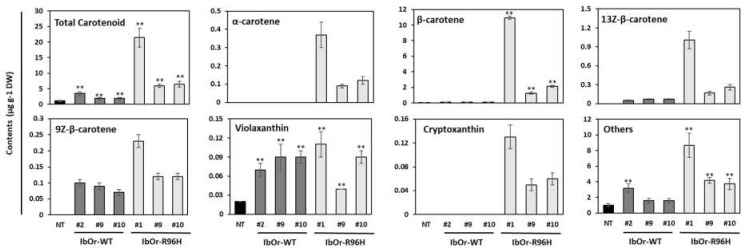
Quantitative HPLC analysis of total carotenoids and individual carotenoid compounds in storage roots of NT and transgenic sweetpotato plants. Data represent mean ± SD of three independent replicates. Significant differences between *IbOr*-*WT*, *IbOr*-*R96H*, and NT plants are indicated with asterisks ** *p* < 0.01; ANOVA, followed by Tukey’s HSD post hoc test).

Previously, overexpression of *IbOr*, *AtOr*, and *SbOr* carrying the golden SNP increased the total carotenoid and β-carotene contents of various plant species. Additionally, *IbOr*-*R96H* transgenic calli accumulated significantly higher levels of total carotenoids and β-carotene than *IbOr*-*WT* transgenic calli, based on the color of the callus [22,31]. To assess whether the color difference correlated with carotenoid accumulation, we analyzed the carotenoid content of storage roots and leaves of NT, *IbOr*-*WT*, and *IbOr*-*R96H* plants by HPLC. The *IbOr*-*R96H* transgenic plants exhibited differences in the carotenoid composition and content of storage roots (Figure 3 and Table S3) and leaves (Table S4) compared with NT and *IbOr*-*WT* transgenic plants. Depending on the transgenic line, the total carotenoid contents of *IbOr*-*R96H* and *IbOr*-*WT* transgenic storage roots were 5.4–19.6- and 1.7–3.2-fold higher, respectively, than that of NT storage roots. Among various carotenoids, β-carotene showed a dramatic overaccumulation in *IbOr*-*R96H* transgenic storage roots; its level was 21.7–186.2-fold higher in *IbOr*-*R96H* transgenic storage roots than in NT storage roots. The amount of α-carotene varied from 0.09–0.37 μg g^−1^ DW in storage roots of the three *IbOr*-*R96H* transgenic lines; however, α-carotene was undetectable in NT and *IbOr*-*WT* transgenic storage roots (Figure 3). Interestingly, β-cryptoxanthin content varied from 0.05 μg g^−1^ DW (line #9) to 0.13 μg g^−1^ DW (line #1) in *IbOr*-*R96H* transgenic storage roots, but was undetectable in NT and *IbOr*-*WT* storage roots (Figure 3 and Table S3). Thus, the promotion of β-carotene accumulation in *IbOr*-*R96H* transgenic plants was consistent with that observed in *IbOr*-*R96H* transgenic sweetpotato calli [31]. In leaves, lutein was the most abundant carotenoid; this result was obtained not only in NT plants but also in transgenic lines (three *IbOr*-*WT* and three *IbOr*-*R96H*). Unlike storage roots, the total carotenoid and β-carotene contents of *IbOr*-*WT* and *IbOr*-*R96H* plants were slightly lower than those of NT plants (Table S4).

### 3.3. IbOr-R96H Overexpression Significantly Altered the Expression of Carotenoid Metabolism-Related Genes

**Figure 4:**
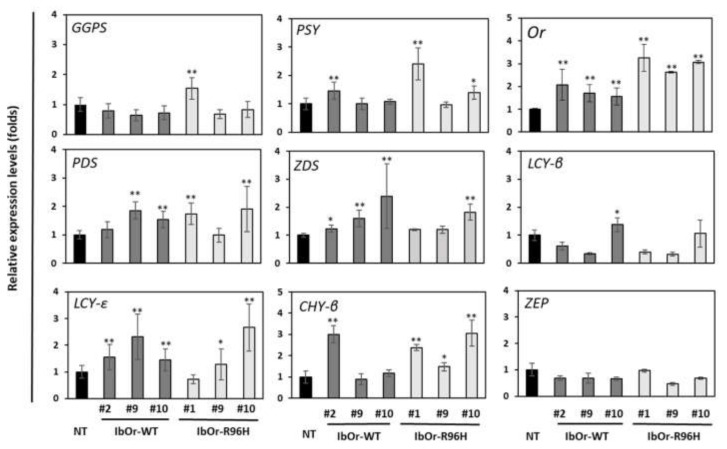
Expression patterns of carotenoid biosynthesis genes in transgenic and NT sweetpotato plants. GGPS, geranylgeranyl pyrophosphate synthase; PSY, phytoene synthase; PDS, phytoene desaturase; ZDS, zeta-carotene desaturase; LCY-β, lycopene β-cyclase; LCY-ε, lycopene epsilon cyclase; CHY-β, beta-carotene hydroxylase; ZEP, zeaxanthin epoxidase. Data represent the mean ± SD of three independent replicates. Significant differences between *IbOr*-*WT*, *IbOr*-*R96H*, and NT plants are indicated with asterisks (* *p* < 0.05, ** *p* < 0.01; one-way ANOVA and Tukey’s HSD post hoc test).

Previously, we showed that most carotenoid biosynthetic pathway genes were up-regulated in the transgenic sweetpotato calli and plants overexpressing *IbOr* [23,31,33]. To determine whether the overaccumulation of carotenoids in storage roots of *IbOr*-*R96H* plants is correlated with altered expression of carotenogenesis-related genes, we analyzed the transcript levels of carotenoid biosynthesis genes in NT, *IbOr*-*WT*, and *IbOr*-*R96H* plants by qRT-PCR. The results showed that the *Or* gene was highly expressed in *IbOr*-*R96H* transgenic storage roots. In addition, *phytoene synthase* (*PSY*), *phytoene desaturase* (*PDS*), *zeta-carotene desaturase* (*ZDS*), *lycopene ε-cyclase* (*LCY*-*ε*), and *beta*-*carotene hydroxylase* (*CHY*-*β*) genes were expressed to higher levels in *IbOr*-*WT* and *IbOr*-*R96H* transgenic storage roots than in NT storage roots (Figure 4).

**Figure 5:**
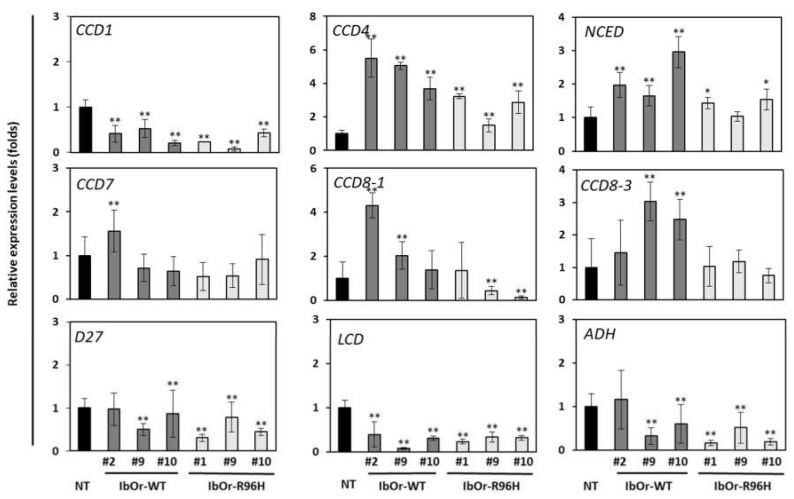
Expression patterns of carotenoid degradation genes in transgenic and NT sweetpotato plants. CCD, carotenoid cleavage dioxygenase; NCED, 9-cis-epoxycarotenoid dioxygenase; D27, carotene isomerase; LCD, lycopene cleave dioxygenase; ADH, aldehyde dehydrogenase. Data represent mean ± SD of three independent replicates. Significant differences between *IbOr*-*WT*, *IbOr*-*R96H*, and NT plants are indicated with asterisks (* *p* < 0.05, ** *p* < 0.01; one-way ANOVA, followed by Tukey’s HSD post hoc test).

We previously showed that expression levels of carotenoid cleavage genes were elevated in *IbOr*-*R96H* transgenic calli compared with NT calli [31]. Therefore, we investigated the transcript levels of carotenoid degradation pathway genes in *IbOr*-*R96H* storage roots. Expression levels of *carotenoid cleavage dioxygenases 4* (*CCD4*) and *9-cis-epoxycarotenoid dioxygenase (NCED)*, which are involved in carotenoid cleavage, were higher in *IbOr*-*WT* and *IbOr*-*R96H* transgenic storage roots than in NT storage roots, consistent with our previous results. However, the transcript levels of these genes were lower in *IbOr*-*R96H* storage roots than in *IbOr*-*WT* storage roots. In addition, the expression levels of carotenoid degradation genes such as *CCD1, carotene isomerase (D27), lycopene cleavage dioxygenase (LCD)*, and *aldehyde dehydrogenase* (*ADH*), which are involved in lycopene and β-carotene cleavage, were lower in *IbOr*-*R96H* transgenic storage roots than in NT and *IbOr*-*WT* transgenic storage roots (Figure 5). These results indicate that enhanced accumulation of carotenoids in *IbOr*-*R96H* transgenic storage roots might be an effect of the lower expression of carotenoid cleavage genes.

### 3.4. IbOr-R96H Transgenic Plants Exhibit Increased Antioxidant Activity and Heat Stress Tolerance

**Figure 6:**
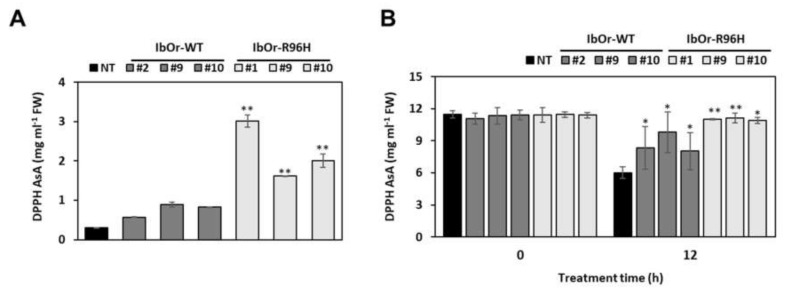
Antioxidant activity in transgenic sweetpotato plants. (**A**,**B**) Analysis of DPPH radical scavenging activity in the storage roots (**A**) and leaves (**B**) of NT, *IbOr*-*WT*, and *IbOr*-*R96H* plants treated with heat stress for 12 h. Data represent mean ± SD of three independent replicates. Significant differences between *IbOr*-*WT*, *IbOr*-*R96H*, and NT plants are indicated with asterisks (* *p* < 0.05, ** *p* < 0.01; one-way ANOVA, followed by Tukey’s HSD post hoc test).

Previously, *IbOr*-*WT* transgenic sweetpotato calli exhibited increased antioxidant activity and salt stress tolerance. Furthermore, *IbOr*-*R96H* transgenic calli also showed enhanced tolerance to salt and heat stresses [31,33]. To investigate the antioxidant activity in transgenic sweetpotato, we analyzed the DPPH radical scavenging activity in fresh storage roots and leaves of NT, *IbOr*-*WT*, and *IbOr*-*R96H* plants (Figure 6). In storage roots, the DPPH radical scavenging activity was 5.5–10.2- and 1.9–3.0-fold higher in *IbOr*-*R96H* and *IbOr*-*WT* transgenic plants, respectively, than in NT plants (Figure 6A). Additionally, in contrast to the normal (no stress) condition, the DPPH activity in *IbOr*-*R96H* leaves was up to 1.85-fold higher than that in NT leaves after 12 h of heat stress treatment (Figure 6B).

**Figure 7:**
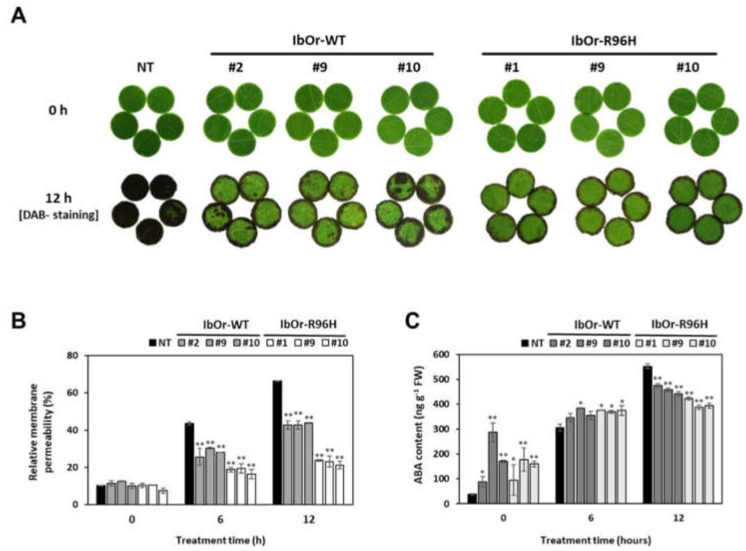
Analysis of heat stress tolerance in transgenic sweetpotato plants using leaf discs. (**A**) Images of leaf discs and quantification of ROS production in leaves using DAB staining. (**B**) Ion leakage from detached leaves treated with heat for 0, 6, and 12 h. (**C**) Quantitative analysis of abscisic acid (ABA) contents in leaves under heat stress. Data represent mean ± SD of three independent replicates. Significant differences between *IbOr*-*WT*, *IbOr*-*R96H*, and NT plants are indicated with asterisks (* *p* < 0.05, ** *p* < 0.01; one-way ANOVA, followed by Tukey’s HSD post hoc test).

Previously, transgenic *IbOr*-*WT* and *IbOr*-*R96H* calli exhibited increased tolerance to heat stress compared with NT calli [31]. To compare the heat stress tolerance of transgenic and NT sweetpotato plants, leaf discs prepared from NT, *IbOr*-*WT*, and *IbOr*-*R96H* plants were incubated at 47 °C for 12 h, and membrane damage was examined by DAB staining and ion leakage analysis. NT plants showed severe membrane damage, as evident from the dark-brown coloration of leaf discs, whereas *IbOr*-*WT* and *IbOr*-*R96H* leaf discs displayed less membrane damage. *IbOr*-*R96H* transgenic plants showed greater heat stress tolerance than NT and *IbOr*-*WT* plants (Figure 7A). After 12 h of heat stress treatment, leaves of NT and *IbOr*-*WT* plants showed 66% and 42.8–43.8% ion leakage, respectively. By contrast, leaves of *IbOr*-*R96H* plants exhibited significantly less ion leakage (21.2–23.75%) at the same time point (Figure 7B). In addition, to investigate the relationship between greater heat stress tolerance and the contents of abscisic acid (ABA), we quantified ABA contents in plants under normal growth conditions and after exposure to heat stress. Under normal and after 6 h of heat stress conditions, ABA contents were higher in *IbOr*-*WT* and *IbOr*-*R96H* plants than in NT plants. This result is consistent with the findings that the expression level of *NCED*, a key enzyme in ABA biosynthesis, was higher in *IbOr*-*WT* and *IbOr*-*R96H* transgenic plants, as shown in Figure 5. After 12 h of heat stress treatment, the highest values of ABA contents were recorded in NT plants compared to *IbOr*-*WT* and *IbOr*-*R96H* plants (Figure 7C). These results suggest that the increases in DPPH radical scavenging activity and ABA contents in *IbOr*-*R96H* plants lead to greater tolerance to heat stress.

## 4. Discussion

Overexpression of the golden SNP-carrying mutant *CmOr* allele resulted in enhanced carotenoid content and orange-colored melon fruit phenotypes [18]. Overexpression of the *IbOr* gene carrying the same mutation resulted in high total carotenoid and β-carotene contents and enhanced abiotic stress tolerance in sweetpotato [31]. In this study, we successfully generated transgenic sweetpotato plants overexpressing *IbOr*-*R96H*. In carrot, the root color depends on the contents of different carotenoids: orange-colored carrot genotypes contain mostly α-carotene and β-carotene; yellow and red carrots contain lutein and lycopene, respectively; and white genotypes contain almost no carotenoid [34]. In storage roots of sweetpotato, the β-carotene content varies greatly with the color of different varieties of sweetpotato, which ranges from white to light yellow, deep yellow, and dark orange [35,36]. Overexpression of *IbOr*-*R96H* in sweetpotato changed the color of storage roots from white to orange (Figure 2). These results suggest that overexpression of *IbOr*-*R96H* increased the contents of carotenoids, especially β-carotene, in storage roots.

Carotenoids play important roles in the primary and secondary metabolism of plants and accumulate to high levels in tissues of some plants. Xylem and phloem surrounding the cambium layers represent secondary tissues in the plant vascular system. Xylem conducts water, while phloem conducts amino acids and sucrose. Interestingly, overexpression of *IbOr*-*R96H* in sweetpotato changed the color of the area surrounding the cambium ring in storage roots (Figure 2B, right). Unlike most crops, the phloem and xylem tissues in carrot roots are visually well separated by the vascular cambium. Previously, some studies showed that the level of carotene was higher in secondary phloem than in secondary xylem of carrot roots. In phloem chromoplasts, carotene is mainly deposited in the crystalline form. Carotenoid metabolites induce structural changes in carotene deposited in chromoplasts, the main organelles of carotenoid accumulation, and crystalline chromoplast development leads to the massive accumulation of carotene pigments [37,38,39,40]. Both carrot and sweetpotato contain high levels of β-carotene and α-carotene. In the carrot root, the proportion of crystalline chromoplasts is the highest compared with other substructures. However, in sweetpotato, chromoplasts exert various substructures such as globular, crystals, and plastoglobuli, together with membrane substructures [41]. Therefore, it is possible that a color change in storage roots of *IbOr*-*WT* and *IbOr*-*R96H* transgenic sweetpotato plants, specifically in the area surrounding the cambium ring, is related to the overaccumulation of carotenoids and structural modification of carotenes.

In addition, storage roots of *IbOr*-*R96H* plants accumulated carotenoids to higher levels than *IbOr*-*WT* and NT plants. Similar to *IbOr*-*R96H* transgenic sweetpotato calli, storage roots of *IbOr*-*R96H* plants showed a dramatic increase in total carotenoid content (up to 19.6-fold) and β-carotene content (up to 186.2-fold) compared with those of NT plants. Additionally, the total carotenoid and β-carotene contents of *IbOr*-*R96H* storage roots were 3.2–6.1- and 11.5–99.3-fold greater than those of *IbOr*-*WT* storage roots, respectively (Figure 3). In contrast to the results obtained with storage roots, the leaves of NT, *IbOr*-*WT*, and *IbOr*-*R96H* plants showed no significant differences in carotenoid contents (Table S4). Unlike non-photosynthetic tissues such as fruits and roots, leaves rarely exhibit an overaccumulation of carotenoids. In plant leaves, carotenoids are continuously synthesized and degraded to maintain optimal photosynthesis through carotenoid steady-state regulatory mechanisms [42,43,44,45]. These results indicate that leaves maintain carotenoid homeostasis, however, through unknown mechanisms.

Carotenoid cleavage reactions play a major role in determining the carotenoid content of plants. Lycopene, β-carotene, and zeaxanthin act as precursors of important apocarotenoids. The conversion of carotenoids into apocarotenoids is catalyzed by *carotenoid cleavage dioxygenases* (CCDs), which target different double bonds of the carotenoid polyene chain. The *9-cis-epoxycarotenoid dioxygenase* (NCEDs) cleave 9-*cis*-violaxanthin and 9-cis-neoxanthin into xanthoxin, and synthesize abscisic acid (ABA), an important hormone involved in plant stress response and seed maturation. The *lycopene cleavage dioxygenase* (LCD) enzyme, along with ADH, converts lycopene into bixin, a polyene. Enzymes including CCD1, D27, LCD, and ADH mediate lycopene and β-carotene cleavage [46,47,48]. We showed that overexpression of *IbOr*-*R96H* in sweetpotato storage roots increased not only the total carotenoid content but also *CCD4* and *NCED* expression levels (Figure 4). In addition, the expression levels of carotenoid degradation genes such as *CCD1*, *D27*, *LCD*, and *ADH* were lower in storage roots of *IbOr*-*R96H* plants than in those of NT and *IbOr*-*WT* plants (Figure 5). These results indicate that the down-regulation of genes encoding carotenoid cleavage enzymes leads to carotenoid overaccumulation in storage roots of *IbOr*-*R96H* plants compared with those of NT and *IbOr*-*WT* plants. Interestingly, we found various expression patterns among *CCDs* in *IbOr*-*R96H* transgenic sweetpotato plants. Especially, we found that there is an inverse effect between *CCD1* and *CCD4*. While expression levels of *CCD1* was lower than in NT storage roots, *CCD4* is highly expressed (Figure 5). Similar to *CCD1*, loss of function in *CCD4* induces carotenoid accumulation in plants. However, in sweetpotato, IbCCD4 interacts specifically with IbOr and its interaction did not affect β-carotene degradation [28]. The relation between CCD4 expression and carotenoid accumulation in *IbOr*-*WT* and *IbOr*-*R96H* plants remains to be studied in detail.

IbOr stabilizes IbPSY, a key regulator of the carotenoid biosynthetic pathway, via its chaperone activity under heat stress. Overexpression of *IbOr* in sweetpotato calli and plants improved tolerance to heat stress [26,30]. Here, we showed that leaf discs excised from *IbOr*-*R96H* transgenic sweetpotato plants displayed greater heat stress tolerance than *IbOr*-*WT* and NT plants. Until 6 h of heat stress conditions, ABA contents were higher in *IbOr*-*WT* and *IbOr*-*R96H* plants than in NT plants (Figure 7). In addition, the DPPH radical scavenging activity, which reflects the antioxidant activity, was higher in *IbOr*-*R96H* plants than in *IbOr*-*WT* and NT plants (Figure 6). Carotenoids as antioxidants can quench singlet oxygen and scavenge superoxide anion radicals. In addition, DPPH is widely used to evaluate the ability of compounds as a free radical scavenger to enhance antioxidant activity [49,50]. Furthermore, ABA is an important isoprenoid stress-induced phytohormone. The ABA is quickly synthesized and is regulated by the ABA biosynthesis *NCED* genes under abiotic stresses such as temperature, drought and salinity [51,52]. In this study, DPPH radical activity and ABA contents with enhanced transcript level of *NCED* in transgenic lines were much higher than those in the NT line. These results suggest that overexpression of *IbOr*-*R96H* in sweetpotato not only increases the contents of total carotenoid and β-carotene but also enhances heat stress tolerance via increased ABA level and DPPH radical scavenging activity. The relation between heat stress tolerance and ABA contents in *IbOr*-*WT* and *IbOr*-*R96H* plants remains to be studied in detail.

To further analyze the regulatory mechanisms of IbOr-R96H, we are currently investigating the potential IbOr-R96H-interacting partners associated with carotenoid overaccumulation and abiotic stress tolerance. Furthermore, we anticipate that site-specific mutagenesis of *IbOr* combined with CRISPR-Cas9-mediated base-editing techniques could be an effective tool for the improvement of carotenoid contents in sweetpotato. Therefore, we are currently under-developing transgenic sweetpotato plants expressing *IbOr*-*R96H*. Overall, the *IbOr*-*R96H* gene could help us cope with global food and nutrition security problems, and help to establish a sustainable society in the face of climate change.

## 5. Conclusions

In this study, we showed that overexpression of *IbOr*-*R96H* significantly increased carotenoid accumulation and antioxidant activity in sweetpotato storage roots to levels higher than those achieved by the overexpression of *IbOr*-*WT*. Storage roots of *IbOr*-*R96H* transgenic sweetpotato plants showed up to 19.6- and 186.2-fold higher total carotenoid and β-carotene contents, respectively, compared with those of NT plants. In addition, gene expression analysis indicated that genes encoding carotenoid cleavage enzymes might be involved in the overaccumulation of carotenoids in *IbOr*-*R96H* storage roots. Furthermore, at 47 °C, *IbOr*-*R96H* transgenic sweetpotato plants displayed greater heat stress tolerance than *IbOr*-*WT* plants, which was associated with higher DPPH radical scavenging activity and ABA contents. Taken together, our results demonstrate that overexpression of *IbOr*-*R96H* in sweetpotato confers heat stress tolerance by triggering the production of carotenoids and improving free radical scavenging capabilities.

## Data Availability

Data is contained within the article.

## Supplementary Materials

The following are available online at https://www.mdpi.com/2076-3921/10/1/51/s1. Table S1: List of primers used for quantitative real-time PCR (qRT-PCR) analysis of sweetpotato genes involved in carotenoid biosynthesis; Table S2: List of primers used for qRT-PCR analysis of sweetpotato genes involved in carotenoid degradation; Table S3: Carotenoid contents of storage roots of transgenic and non-transgenic sweetpotato plants; Table S4: Carotenoid contents of leaves of transgenic and non-transgenic sweetpotato plants.
