# Supplementary material for: Overexpression of the Golden SNP-Carrying Orange Gene Enhances Carotenoid Accumulation and Heat Stress Tolerance in Sweetpotato Plants
Source: Antioxidants (Basel). 2021 Jan 4;10(1):51. doi: 10.3390/antiox10010051 (PMC7823567; doi:10.3390/antiox10010051)
Supplement: Supplementary file 1 [file antioxidants-10-00051-s001.pdf]

Supplemental Table S1. Primer sequences for genes involved in carotenoid biosynthesis in sweetpotato

Target gene	Forward (5'-3')	Reverse (5'-3')	Application
IbOr	GCACTGGATCACTAGTCCTT	GTCAATTCGTGGGTCATGCT	Real-time PCR
GGPS	TAAGACGGAGAGCGTGGAGA	TGGGATGACCTTATCACCAT	Real-time PCR
PSY	AAGTTCTTCGACGAGGCTGA	GCAGTTTCTTTGGCTTGCTT	Real-time PCR
PDS	GTACAAAACCGTGCCAGGAT	TCCTTTAAAATCGCCTGTGC	Real-time PCR
ZDS	ATAGCATGGAAGGAGCAACG	AGCTCATCAGATACAGCAGCAG	Real-time PCR
LCY-β	ATGGTGTGACGATTCAAGCA	GCCAATCCATGAAAACCATC	Real-time PCR
LCY-ε	ACCAGTTGGAGGATCATTGC	CACCCATACCAGGACTTCGT	Real-time PCR
CHY-β	CAAGAGAAGGACCGTTCGAG	GACGAACATGTAGGCCATCC	Real-time PCR
ZEP	TGGTACTTGGATCACCGACA	GCTGCCTGCAAAACTTTCAT	Real-time PCR
UBI	TCGACAATGTGAAGGCAAAG	CTTGATCTTCTTCGGCTTGG	Real-time PCR

Supplemental Table S2. Primer sequences for genes involved in carotenoid degradation pathway in sweetpotato

Target gene	Forward (5'-3')	Reverse (5'-3')	Application
CCD1	CGGTGGAGAAACTCACGATT	TCCCGATATCTTCGGCATAG	Real-time PCR
CCD4	TGCTGAACCAGAGACTGGAA	TCCGGTGTTCTCATCGTGTA	Real-time PCR
NCED	GGGAAGATCCCGGAGTGTAT	GGACCAATCTATGCGTCTCC	Real-time PCR
CCD7	GGCGAGGTACATTCGGACGG	TCGCCACCTTCCCAGAGACA	Real-time PCR
CCD8-1	AACACCGGCGTCGTCAAACT	CATCCTCGCCACGACGTACC	Real-time PCR
CCD8-3	GCGCAAAATCTGCTCAGGGC	GTTGTGCTCGCAGCAATCGG	Real-time PCR
D27	TGGTCCAGGAGTCGGGATGG	TACCATCACGGCCGCAACTC	Real-time PCR
LCD	CGTCATCACTCTCGGCCGTC	CGGGACGTCGGGTTGTTTGA	Real-time PCR
ADH	GGGGACGTTGCGGTACTACG	ACAATTGTGCAACCTGCGGC	Real-time PCR
UBI	TCGACAATGTGAAGGCAAAG	CTTGATCTTCTTCGGCTTGG	Real-time PCR

Supplemental Table S3. Carotenoid contents in storage roots

	violaxanthin		cryptoxanthin		13Z-β-carotene		α-carotene		β-carotene		9Z-β-carotene		others		Total	
**NT**	**0.02**	**±0.00**	**ND**		**ND**		**ND**		**0.06**	**±0.01**	**ND**		**1.01**	**±0.19**	**1.09**	**±0.20**
**WT#2**	**0.07**	**±0.01**	**ND**		**0.05**	**±0.01**	**ND**		**0.11**	**±0.02**	**0.10**	**±0.01**	**3.18**	**±0.55**	**3.52**	**±0.60**
**WT#9**	**0.09**	**±0.02**	**ND**		**0.07**	**±0.01**	**ND**		**0.11**	**±0.02**	**0.09**	**±0.01**	**1.56**	**±0.29**	**1.92**	**±0.34**
**WT#10**	**0.09**	**±0.01**	**ND**		**0.07**	**±0.01**	**ND**		**0.11**	**±0.02**	**0.07**	**±0.01**	**1.55**	**±0.30**	**1.88**	**±0.36**
**R96H#1**	**0.11**	**±0.02**	**0.13**	**±0.02**	**1.01**	**±0.14**	**0.37**	**±0.07**	**10.92**	**±1.19**	**0.23**	**±0.02**	**8.66**	**±1.55**	**21.44**	**±3.01**
**R96H#9**	**0.04**	**±0.00**	**0.05**	**±0.01**	**0.17**	**±0.03**	**0.09**	**±0.01**	**1.27**	**±0.14**	**0.12**	**±0.01**	**4.18**	**±0.38**	**5.93**	**±0.58**
**R96H#10**	**0.09**	**±0.01**	**0.06**	**±0.01**	**0.26**	**±0.04**	**0.12**	**±0.02**	**2.14**	**±0.13**	**0.12**	**±0.01**	**3.74**	**±0.72**	**6.53**	**±0.94**

Supplemental Table S4. Carotenoid contents in leaves

	violaxanthin	lutein	zeaxanthin	cryptoxanthin	13Z-β-carotene	α-carotene	β-carotene	9Z-β-carotene	others	Total
**NT**	268.0±50.8	1527.5±64.1	58.2±5.2	0.08±0.00	55.7±1.5	149.5±10.8	801.2±65.1	126.0±14.6	275.6±40.5	3261.8±252.7
**WT#2**	266.8±30.4	1557.0±40.6	53.7±6.6	0.07±0.00	42.7±5.7	158.6±8.9	708.2±42.4	118.4±9.6	251.3±13.5	3156.8±157.7
**WT#9**	295.0±36.0	1526.5±69.2	30.0±5.7	0.07±0.00	43.6±6.0	145.0±13.5	703.2±68.9	110.8±11.6	250.2±23.0	3104.4±234.0
**WT#10**	242.1±31.1	1401.4±67.8	50.6±4.0	0.07±0.00	41.5±6.6	122.9±9.8	622.6±28.4	101.6±5.2	244.7±27.6	2827.6±180.5
**R96H#1**	287.7±33.3	1732.6±95.4	58.6±2.0	0.07±0.00	52.8±5.1	209.5±13.3	777.3±39.9	124.6±8.5	250.4±43.8	3493.6±241.2
**R96H#9**	214.2±6.0	1516.1±155.1	57.6±8.4	0.07±0.00	46.6±7.1	189.1±24.5	670.7±71.7	111.5±12.7	231.5±15.0	3037.4±300.5
**R96H#10**	242.5±43.8	1450.0±170.1	52.8±5.0	0.07±0.00	44.9±6.5	157.4±16.8	614.8±61.3	119.4±18.3	278.5±49.0	2960.4±370.8
